# Exploration of the psychometric properties of the Clinical Outcomes in Routine Evaluation-Outcome Measure in Ecuador

**DOI:** 10.1186/s40359-020-00443-z

**Published:** 2020-09-01

**Authors:** Clara Paz, Guido Mascialino, Chris Evans

**Affiliations:** 1 School of Psychology, Universidad de Las Américas, Jose Queri y Av. De los Granados, EC170125 Quito, Ecuador; 2 Department of Psychology, The University of Sheffield, Cathedral Court, 1 Vicar Lane, Sheffield, S1 2LT UK

**Keywords:** CORE-OM, Outcome measure, Psychometric properties, Latin America, Psychological distress

## Abstract

**Background:**

The Clinical Outcomes in Routine Evaluation-Outcome Measure (CORE-OM) is a pan-theoretical and pan-diagnostic measure of mental health designed to cover issues that people wish to change in psychotherapy. The objective of this study was to explore the psychometric properties of the Spanish translation of the CORE-OM, in a country, Ecuador for which there is not a single measure suitable for this purpose with empirically demonstrated local acceptability and psychometric properties.

**Methods:**

In total, 886 adults not currently receiving psychotherapy treatment or taking psychotropic medication were included in the analysis. The analyses broadly followed and compared with results from previous studies. These analyses consisted of assessment of acceptability, internal consistency, test-retest reliability, influences of demographic variables, correlations between domain scores, and convergent validity with Spanish versions of the Outcome Questionnaire 45.2 and Schwartz Outcome Scale-10.

**Results:**

The questionnaire showed good acceptability (overall omission rate of 0.56%), good reliability (*α* = .93 [.92, .94], test-retest correlations ranged from .59 to .85), and good convergent validity with the Outcome Questionnaire 45.2 (*r* = .84) and the Schwartz Outcome Scale-10 (*r* = −.73). Statistically significant gender differences were found in two domains: females scored higher on Well-being (*M* = 1.23) than males (*M* = 1.01), though effect size was small (*g* = 0.31); and males (*M* = 0.31) scored higher than females on Risk (*M* = 0.25), with even smaller effect size (*g* = 0.06). Age was negatively correlated with psychological distress in all domains and coefficients ranged from −.14 for Risk to −.29 for Functioning.

**Conclusions:**

The results support the use of the CORE-OM as a valid and reliable instrument in a non-clinical Ecuadorean population. Exploration of the psychometric properties in a clinical population is recommended to assure its use in clinical settings.

## Background

Increasing recognition of the substantial burden of disease created by mental health disorders [1] across all regions of the world has underscored the need for measures that allow comparison of different interventions across geographic regions, multiple clinical settings, and varying disorders. Over the last few decades, research on psychological interventions has increased, in large part through efforts to improve the efficacy and effectiveness of interventions [2]. However, this work has been very unevenly distributed across countries and the world’s population.

Growth in this area requires research into the change/outcome measures needed by both researchers and clinicians to assess treatments. However, outcome measure development has been fragmented and largely dominated by symptom-specific, or setting-specific, instruments with few scales designed to measure general outcomes across varied settings [3]. Exacerbating this, research into, and adoption of, outcome measurement outside developed countries is even lower [4] than in developed countries.

The Clinical Outcomes in Routine Evaluation-Outcome Measure (CORE-OM) was designed to be a pan-theoretical, pan-diagnostic measure of mental health focused on issues people wish to change in therapy. It emerged from qualitative and quantitative work with clinicians and service users about what needs to be measured regarding psychological well-being and change in psychotherapy [5, 6]. The resulting 34-item self-report measure has been very widely used in clinical practice and psychotherapy research across different types of therapies, clinical settings, and symptomatology. It was intended to cover four conceptual domains (Well-being, Problems, Functioning and Risk) though these were never expected to represent any clean population factor structure. Though copyright to CORE Systems Trust (CST), the CORE-OM has always been free of reproduction fees and is available under a Creative Commons license so it can be downloaded freely [7].

As intended by the designers, the CORE-OM has been utilized in research for many purposes, such as determining the level of psychological well-being in a given population [8], evaluating the effect of psychological interventions [9, 10], exploring psychotherapy process [11], and as an outcome measurement in randomized controlled trials [12]. The CORE-OM has been also used to generate practice-based evidence, a paradigm that looks for complementing evidence-based practice through the provision of information recovered for practitioners everyday practice [9]. The measure was made available free of reproduction costs to support its use whether in large mental health services, but also to ensure that it could be used in small services or private practice. The information collected using CORE-OM by practitioners can serve for many purposes, one of them as a feedback system of the progress of their clients [13].

Whilst not expected to transfer without any changes of meaning, of psychometrics or of referential score distributions across all cultures and languages, the CORE-OM was hoped to transfer across many and it has now been translated into over 25 languages [14]. All these translations have followed the CST protocol [14] and respected the philosophy to offer translations which might be acceptable to very diverse patients/clients. Psychometric properties in the original UK exploration were good [5]. Internal consistency ranged from acceptable to excellent (*α* = .75 to .94), test-retest reliability was excellent (*ρ =* .91), and convergent validity was good as evidenced by strong correlations with the Beck’s Depression Inventory-II [15] (*r* = .85) and the Symptom Checklist 90-Revised [16] (*r* = .88). Similar explorations in Portuguese [17] and Icelandic [18] versions have shown comparable psychometric properties to the original UK English version [5]. The psychometric properties of the Spanish version [19] were also good. Analysis revealed acceptable to excellent internal consistency (*α* range = .73–.94), adequate to good test-retest reliability (*ρ* = .76–.87) except for the Risk domain (*ρ* = .45), and good convergent validity with the Beck’s Depression Inventory-II [15] (*r* = .83) and the Symptom Checklist 90-Revised [16] (*r* = .79).

As is common, the Spanish translation was developed and validated solely in Spain though there are 21 countries in which Spanish is the official language [20]. The objective of this study is to explore the psychometric properties of the Spanish translation of the CORE-OM [19] in Ecuador, a country in which there is, as far as we have been able to determine, not a single measure suitable for assessment of change in psychological therapies with empirically demonstrated acceptability and psychometric properties [21].

## Methods

### Procedures

This is a psychometric exploratory study that aims to evaluate the psychometric properties of the Spanish translation of the CORE-OM [19] in Ecuador in a non-clinical sample. Data collection occurred from December 2017 to May 2018. Participants were excluded if they reported receiving psychotherapy treatment and/or if they were taking psychotropic medication. They were excluded as they can be considered a clinical population and the immediate focus of the study pending the accumulation of a clinical sample was on the properties of the measure in the non-clinical population. The total sample consisted of two subgroups: a student subsample and a community subsample as in previous studies [5, 19]. Having those two subsamples allow the comparison of the properties with those studies as, though easier to recruit, student samples are clearly not representative of the entire non-clinical population. Convenience sampling was used given low funding and the exploratory nature of the study. The sample size calculation took into consideration the various analyses planned (of acceptability, internal reliability, convergent correlations, age and gender effects and test-retest stability in the student subsample). Minimum sample sizes to give good power to detect meaningful differences from the Spanish [19] findings varied across those analyses. The key analysis with the lowest power was comparison of completion rates for acceptability. This would have a 95% confidence interval from .93 to .96 around an observed completion rate of .95 for a sample of 700, which seemed sufficiently precise for comparison with existing findings. For the test-retest study a sample of 100 would give power to detect the key time 2 to time 3 mean change, which was expected to be small. Data collection was planned to continue until the study period was completed with the period based on resources but which, allowing for a high estimated refusal rate would we thought guarantee the minimum sample sizes being exceeded. In the event better than expected recruitment this resulted in larger than minimal samples.

Student participants were recruited from a private university. They were approached in their classrooms and invited to participate in the study. Participation was voluntary and students received no extra credit. The student participants completed the retest at three time points, each two weeks apart. The community subsample was recruited by snowball sampling starting from the student participants who were asked to inform relatives, friends and work colleagues of the study and to provide the researchers with contact details of those interested in participating. Three members of the research team contacted the potential participants and informed consent was obtained from those willing to participate. A member of the research team was present with the participant until they completed the research measures and forms. Participants were enrolled until the planned termination of recruitment, but recruitment was stopped earlier for some gender/age groups (18 to 30, 31 to 43, and older than 44 years of age) to achieve near balanced group sizes.

### Measures

**CORE-OM** [5, 6] is a self-report questionnaire of 34 items covering four domains: Well-being (four items), Problems/Symptoms (12 items), Functioning (12 items), and Risk (six items). The CORE-OM authors recommend that the domain scores were for a possible utility where a client had problems mainly in one domain. The items are scored on a five-point Likert scale from 0 (“Not at all”) to 4 (“Most or all of the time”). Higher scores indicate higher levels of psychological distress. As it is indicated in the introduction, exploration of the psychometric properties of a number of the translations (e.g. [17–19]) has shown good psychometric properties comparable to the original UK English version.

For this study, a comprehensive procedure was conducted to explore whether the Spanish version of the CORE-OM is understandable to an Ecuadorean Spanish-speaker. Initially, interviews were conducted with 11 people forming a purposive sample designed to cover the four main regions and dialects: Coastal, Andean, Amazonia, and Galápagos. Moreover, this sample did not included participants with higher education. Using a “talk aloud” mode, participants were asked to read an item aloud and paraphrase or explain it. Then, they were asked: a) if they found the item understandable and b) if they found it appropriate for the Ecuadorean context. All interviewed participants considered 33 of the 34 items understandable and appropriate for the Ecuadorean population. The one concern was that item 27 (English version: “I have felt unhappy,” Spanish version: “Me he sentido infeliz”) was understandable but might not be appropriate because the word “infeliz” is not commonly used in Ecuador. On further questioning, participants reported that the word “triste” might be more appropriate to capture unhappiness. As a result, a new version of the item was developed using that word: “me he sentido triste.” In order to evaluate whether this difference would have an effect on responding, a 35-item version was used in this study with either “me he sentido infeliz” or “me he sentido triste” as item 27 and the other version as item 35 with balanced random order. A manuscript describing in detail the methodology and rationale for this linguistic adaptation has been recently submitted for publication.

The Outcome Questionnaire (OQ 45.2) [22] is a 45-item self-report questionnaire, designed to monitor treatment outcomes in mental health settings. It contains three subscales: Symptom Distress (SD), Interpersonal Relations (IR), and Social Role (SR). Items are rated on a five-point Likert scale that ranges from 0 to 4. Both the original version in English [22] and the Chilean Spanish version [23] have demonstrated acceptable psychometric properties. The OQ-45 and CORE-OM had very similar design aims though both teams were unaware of the other team’s work until publication. The OQ-45 is not copyleft, so is not free to use without payment of a licence fee, a disadvantage in a country where mental health interventions are not well funded. However, its similar content and function made it a good choice for convergent validity exploration for the CORE-OM.

**Schwartz Outcome Scale-10 (SOS-10)** [24] was chosen as a second convergent validity check as it is another brief self-report scale with similar aims to the CORE-OM and has a Spanish translation with some psychometric exploration. It has 10 items that measure psychological health and well-being. SOS-10 is free of charge for practitioners, researchers and non-profit health organizations [25]. The scale has shown satisfactory psychometric properties both for the original English version [24] and for the Spanish version [26]. The latter was validated in South Florida, United States, with foreign-born bilingual Spanish-English speakers.

### Analyses

The analyses broadly followed those for the UK version [5] and the Spanish version [19]. They consisted of a) assessment of acceptability, b) internal consistency (Cronbach alpha), c) test-retest reliability, d) influences of demographic variables, e) correlations between domain scores, and f) convergent validity with Spanish versions of the OQ-45.2 and SOS-10. In addition, referential score distribution data are reported. The analyses were conducted for each domain, the total score, and the Non-risk items (i.e. the 28 items not in the risk domain). The use of the Non-risk score is based on the finding, now across all psychometric analyses and languages, that the non-risk items form a large first component with the Risk items tending to be less correlated with those 28 items, making the Non-risk score a slightly more factorial clean score than the total score.

Analyses were exploratory and descriptive. Bootstrapped 95% confidence intervals around sample statistics, including observed effect sizes, were reported rather than *p*-values wherever possible. The a priori analysis of test-retest reliability combined the Spearman correlation, for comparability with earlier studies both in the UK and Spain with exploration of mean change. For the mean change we have reported both Cohen’s *d1* and Cohen’s *dz* to allow for comparisons with other studies which may have reported one but not the other due to a lack of consensus in the literature. Cohen’s *d1* is the mean change divided by the standard deviation (*SD*) of the baseline values, and *dz* is the mean change divided by the *SD* of the change values. Where, as is typical in test-retest studies, there is a strong positive correlation between first and second scores, *dz* will be larger than *d1*. At the request of a reviewer the Intraclass Correlation Coefficient (ICC) has been added to the Spearman's correlation and mean shift analyses. The ICC gives a test-retest statistic penalizing both for mean shift (technically invalidity) and imperfect correlation of measures (unreliability). The coefficient reported is the single rating, random rater, agreement ICC. No internal structure analyses were conducted (in line with Trujillo et al. [19]). This respects the arguments in Evans that the CORE-OM was never intended to have a domain based factor structure but to have wide coverage of many issues, covering the four domains of well-being, problems, functioning and risk which are complexly interrelated both across individuals and in patterns of change within individuals in therapy. This is congruent with the findings of the expected complexity of structure in Lyne et al. [27] and the detailed work of Mavranezouli et al. [28, 29] showing how the complex structure supports health economic evaluation underlining that conventional cross-sectional psychometric structure neatness can be a disadvantage for short, broad coverage measures designed for evaluation of change as well as state at single time points. All analyses were conducted using R version 3.5.1 [30]. The contrast of *“triste”* and *“infeliz”* (item 27) noted above showed no advantage to “triste” and is not reported in detail as here as the extensive exploration of this issue is being reported separately. Given the sensitive nature of the data and non-zero possibility of jigsaw deanonymization the data have not been placed in a public repository but are available from the corresponding author on acceptance of a confidentiality protection agreement.

## Results

### Demographics

**Fig. 1:**
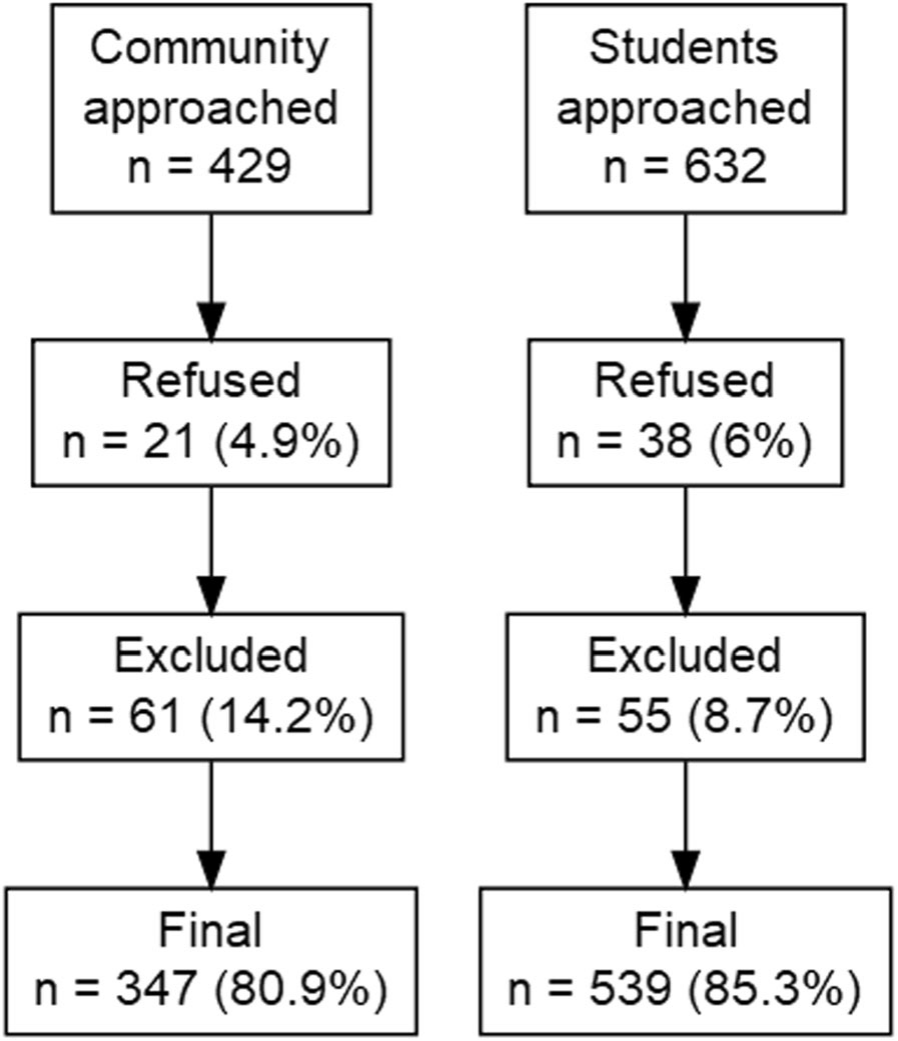
Flow diagram of participants in the study by subsample

Of the 1061 persons invited to participate, 587 were female (55.3%); gender was missing for three (0.3%). After refusals and exclusions, the sample consisted of 886 persons (Fig. 1), 479 of whom were female. The slight excess of females excluded/refusing was not statistically significant (*X*^2^(1) = 3.002, *p* = .08). The female to male ratio was higher in the student sample (58.6%) than in the community sample (47.5%; *X*^2^(1) = 9.84, *p* = .002).

The ages ranged from 18 to 79 (*M* = 28.99, *SD* = 11.89). In the community, 1.4% of the participants reported completing elementary school (6 years), 22.0% had completed high school (12 years), 65.4% reported more than 12 years of education, and for 11.2% this data was missing. Of the student sample, 49.0% were psychology majors, 50.8% non-psychology majors, and for 0.2% this information was missing.

### Acceptability

Of the 886 participants, 811 (91.5%) completed all 34 CORE-OM items, five completed fewer than 31 items leaving 881 (99.4%) participants whose item data could be prorated for an overall score. The overall omission rate across the total sample was 0.56%: items 3 (1.4%), 18 (1.13%), and 17 (1.13%) were the most omitted.

### Internal consistency

Cronbach’s alpha was used to evaluate the internal consistency for each domain, Non-Risk items, and for all items. All analysis showed acceptable levels of internal consistency, although alpha was lowest for the Well-being domain (Table 1).

**Table 1:** Alpha coefficients [95% confidence interval]expressing internal consistency for the Ecuador subsamples, Spain, and United Kingdom samples

Domains	Students (*n* = 344)	Community (*n* = 537)	Pooled sample (*n* = 881)	Trujillo et al.^**a**^ (*n* = 452)	Evans et al.^**b**^ (*n* = 1084)
Well-being	.74[.70, .78]	.51[.42, .61]	.69[.64, .73]	.80 [.77, .83]	.77 [.75, .79]
Problems/Symptoms	.86[.85,.88]	.83[.80, .87]	.86[.85, .88]	.88 [.86, .90]	.90[.89, .91]
Functioning	.83[.81 .86]	.78[.74, .82]	.83[.81, .85]	.86 [.84, .88]	.86[.85, .87]
Risk	.75[.70, .80]	.65[.54, .79]	.73[.68, .78]	.71[.66, .75]	.79[.77, .81]
Non-risk items	.93[.92, .94]	.89[.87, .91]	.92[.91, .93]	.94[.93, .95]	.94[.93, .95]
All items	.94[.93, .94]	.89[.88, .92]	.93[.92, .94]	.94[.93, .95]	.94[.93, .95]

^a^Reproduced with permission from Trujillo et al. (2016). Psychometric properties of the Spanish version of the Clinical Outcomes in Routine Evaluation - Outcome Measure. *Neuropsychiatric Disease and Treatment*, *12*, 1457–66. doi: 10.2147/NDT.S103079

^b^Reproduced with permission from Evans et al. (2002). Towards a standardised brief outcome measure: Psychometric properties and utility of the CORE–OM. *British Journal of Psychiatry*, *180*(1), 51–60. doi: 10.1192/bjp.180.1.51

**Fig. 2:**
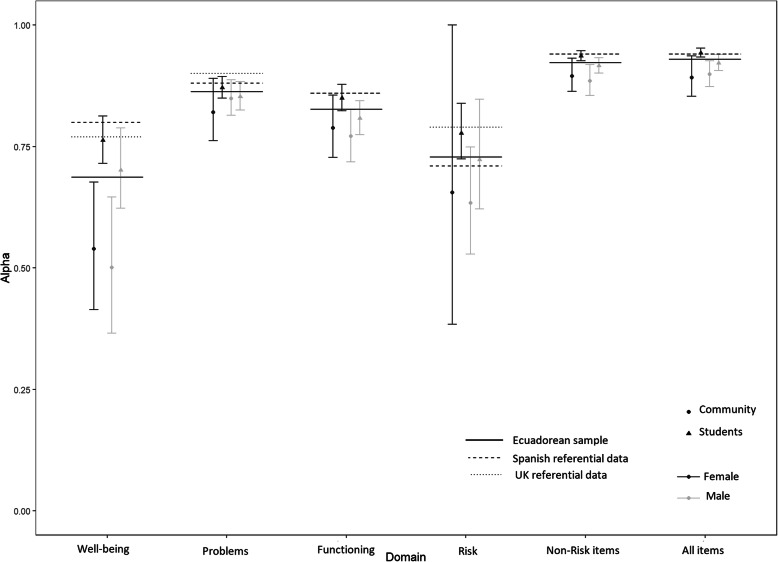
Plot showing comparison between Ecuadorean alpha scores and Spanish and UK referential data

As shown in Fig. 2, there are significant differences in alpha values both between the three countries and between the two Ecuadorean subsamples. Community participants had lower alpha values than students for all domains, but markedly so for the Well-being domain.

### Test-retest stability

**Fig. 3:**
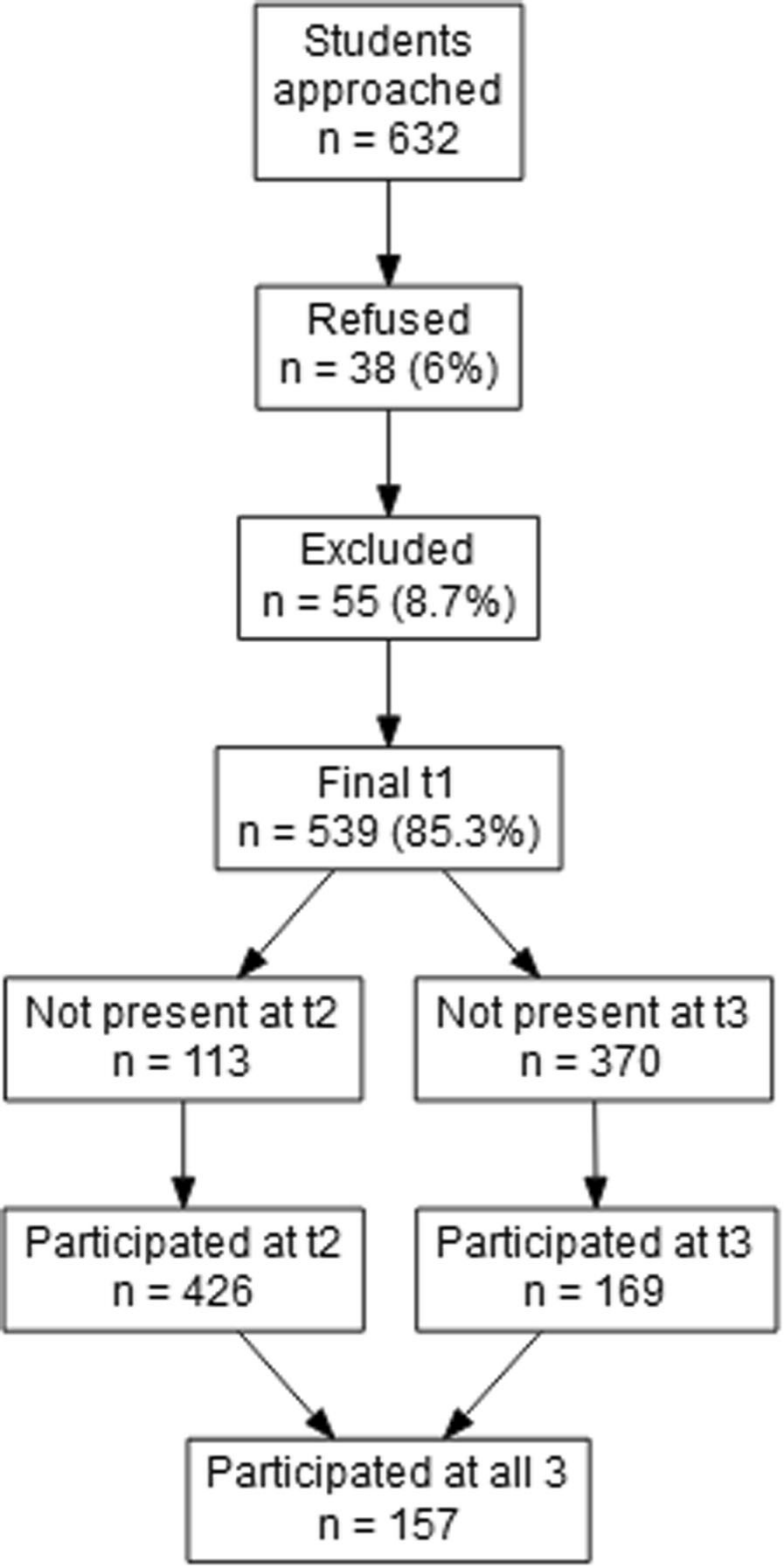
Flow of the students that participated in the retest

The test-retest stability was assessed in the student subsample across three time points (Fig. 3). Test-retest reliability was good for the overall scores and acceptable to good for all the domains (*ρ* = .73–.85), except for the Risk domain (Table 2). All test-retest correlations between the three time points were statistically significant with lower confidence limits well above zero.

**Table 2:** Spearman’s correlation and Intraclass correlation coefficients for first – second and second - third time’s survey for the student subsample denoting test-retest stability

Domains	T1-T2		T2 -T3	
	Spearman’s correlation [95%CI] ^a^	Intraclass Correlation Coefficient [95%CI] ^b^	Spearman’s correlation [95%CI] ^a^	Intraclass Correlation Coefficient [95%CI] ^b^
Well-being	.73 [.68, .78]	.72 [.66, .77]	.78 [.71, .84]	.77 [.70, .83]
Problems/Symptoms	.78 [.74, .82]	.78 [.73, .82]	.76 [.67, .82]	.74 [.65, .80]
Functioning	.82 [.78, .85]	.82 [.78, .85]	.83 [.76, .88]	.82 [.76, .86]
Risk	.61 [.53, .68]	.72 [.66, .77]	.59 [.46, .71]	.58 [.47, .68]
Non-risk items	.84 [.80, .87]	.83 [.79, .87]	.85 [.78, .90]	.83 [.77, .87]
All items	.85 [.81, .88]	.84 [.80, .87]	.85 [.78, .90]	.82 [.77, .87]

^a^Spearman’s rank correlation coefficient with 95% bootstrapped confidence interval. T1 = time 1 (assessment at baseline),T2 = time 2 (assessment 2 weeks after baseline),T3 = time 3 (assessment 4 weeks after baseline)

^b^Single rating, random rating, agreement Intraclass Correlation Coefficient with parametric 95% confidence interval

Stability was also assessed by testing the mean shift between each time point using the Wilcoxon test. There was a statistically significant shift, with small effect sizes, for all scores between time 1 (T1) and time 2 (T2). The shift was not significant from t2 to time 3 (T3) for all domains except Problems/Symptoms, for which a small effect size was found (Table 3). As mentioned above, given the strong positive correlations of scores over the time intervals, the Cohen’s *dz* effect size values were larger than the *d1* values.

**Table 3:** Test-retest stability showing mean values and shift between first, second and third survey time in the student sample

Domains	Time	Mean difference^**a**^ (SD)	95% Bootstrapped CI	Cohen’s d1^**b**^[95% Bootstrapped CI]	Cohen’s dz^**c**^[95% Bootstrapped CI]
Well-being	2–1	− 0.13(0.58)	[− 0.19, − 0.07]	0.17[0.1, 0.24]	0.22[0.13, 0.33]
Problems/Symptoms	2–1	− 0.09(0.44)	[− 0.13, − 0.05]	0.14[0.07, 0.2]	0.20[0.11, 0.31]
Functioning	2–1	− 0.06(0.35)	[− 0.09, − 0.03]	0.11[0.05, 0.17]	0.17[0.07, 0.28]
Risk	2–1	− 0.06(0.31)	[− 0.09, − 0.03]	0.15[0.08, 0.22]	0.20[0.11, 0.3]
Non-risk items	2–1	−0.08(0.34)	[− 0.12, − 0.05]	0.14[0.08, 0.2]	0.24[0.14, 0.34]
All items	2–1	−0.08(0.30)	[− 0.11, − 0.05]	0.15[0.1, 0.2]	0.26[0.16, 0.37]
Well-being	3–2	0.01(0.58)	[−0.09, 0.10]	−0.01[− 0.11, 0.1]	−0.01[− 0.18, 0.16]
Problems/Symptoms	3–2	− 0.11(0.50)	[− 0.19, − 0.03]	0.15[0.04, 0.25]	0.22[0.07, 0.37]
Functioning	3–2	− 0.03(0.39)	[− 0.09, 0.03]	0.05[− 0.05, 0.14]	0.08[− 0.08, 0.25]
Risk	3–2	−0.02(0.32)	[− 0.07, 0.03]	0.06[− 0.11, 0.19]	0.06[− 0.08, 0.24]
Non-risk items	3–2	−0.06(0.38)	[− 0.12, 0.00]	0.09[0.01, 0.18]	0.16[0.01, 0.32]
All items	3–2	−0.05(0.34)	[−0.11, 0.00]	0.09[0, 0.18]	0.16[0, 0.33]

*SD* Standard deviation, *CI* Confidence interval

^a^The Wilcoxon test was used to test ¿the mean shifts between time points

^b^Mean change divided by the standard deviation of the baseline values

^c^Mean change divided by the standard deviation of the change values

### Convergent validity

Convergent validity was assessed by testing the correlations between CORE-OM total and domain scores with SOS-10 and OQ.45.2 total scores (Table 4). Results showed statistically significant and moderate to strong correlations for all the domains. As expected, the correlations with SOS-10 were negative, as higher scores on this measure represent lower levels of psychological distress. The Risk domain showed the lowest correlations (SOS-10 = − .43, OQ-45.2 = .52). University students’ CORE-OM scores showed higher correlations with both SOS-10 and OQ-45.2 than those of the community participants.

**Table 4:** Mean, standard deviations, internal consistency and correlations of SOS-10 and OQ-45.2 with each domain of the CORE-OM, denoting convergent validity

Samples	***M (SD)***	***α***[95% CI]	Domains					
			Well-Being	Problems/Symptoms	Functioning	Risk	Non-risk items	All items
			***r***[95% CI]^**a**^	***r***[95% CI]^**a**^	***r***[95% CI]^**a**^	***r***[95% CI]^**a**^	***r***[95% CI]^**a**^	***r***[95% CI]^**a**^
Students								
SOS-10	46.70(9.61)	.93[.91, .95]	−.73[−.77, −.67]	−.70[−.75, −.64]	−.78[−.81, −.71]	−.54[−.50, −.37]	−.81[−.83, −.74]	−.81[−.82, −.73]
OQ-45.2	53.25(20.42)	.86[.83, .89]	.76[.71, .81]	.82[.78, .86]	.79[.73, .83]	.54[.46, .61]	.87[.84, .90]	.88[.84, .90]
Community								
SOS-10	50.49(8.71)	.92[.90, .93]	−.56[−.65, −.46]	−.49[−.58, −.39]	−.59[−.68, −.49]	−.40[−.49, −.30]	−.62[−.70, −.52]	−.63[−.71, −.53]
OQ-45.2	44.39(17.47)	.90[.88, .91]	.59[.48, .68]	.70[.61, .78]	.62[.51, .71]	.47[.36, .56]	.76[.68, .82]	.76[.68, .82]
Pooled								
SOS-10	48.19(9.44)	.92[.91, .94]	−.68[−.72, −.63]	−.63[−.68, −.58]	−.71[−.75, −.66]	−.43[−.48, −.37]	−.73[−.77, −.69]	−.73[−.77, −.69]
OQ-45.2	49.59(19.73)	.89[.88, .91]	.71[.66, .76]	.79[.75, .82]	.74[.69, .78]	.52[.45, .57]	.84[.80, .87]	.84[.81, .87]

*CI* Confidence interval

^a^ 95% Confidence intervals with Holm’s correction for multiple tests

### Sex, age and education differences

**Fig. 4:**
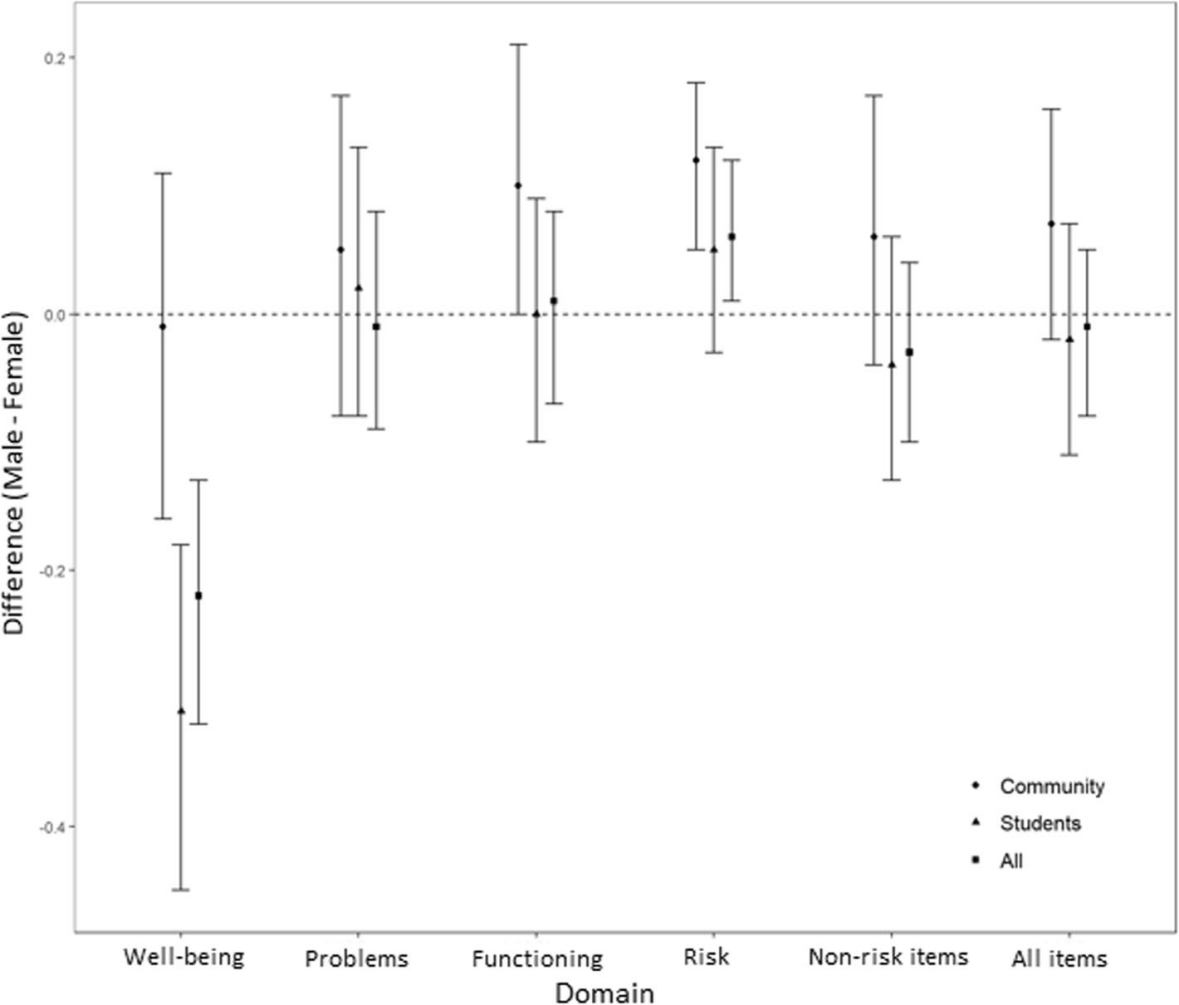
Gender mean differences plot by subsample

Analysis of sociodemographic variables revealed gender differences in some domains but not in overall score (Fig. 4). Across the whole sample, females demonstrated higher scores in the Well-being domain (*M* = 1.23) than males (*M* = 1.01), indicating lower levels of psychological well-being, though effect size was small (*g* = 0.31). While males (*M* = 0.31) had higher scores in the Risk domain than females (*M* = 0.25), this difference had an even smaller effect size (*g* = 0.06). Analyses of gender differences within each subsample showed that female students had a significantly higher score than male students on the Well-being domain (*g* = 0.41) while community males had a significantly higher score on the Risk domain than community females with small effect size (*g* = 0.37). Table 5 contains mean, standard deviations, maximum score and 95th percentile by gender and subsample.

**Table 5:** Mean, standard deviations, maximum score and 95th percentile split by gender and subsample

Domains	Students						Community					
	Male			Female			Male			Female		
	Mean (SD)	95th percentile	Max	Mean (SD)	95th percentile	Max	Mean (SD)	95th percentile	Max	Mean (SD)	95th percentile	Max
Well-being	1.07(0.73)	2.50	3.50	1.38(0.77)	2.75	4.00	0.93(0.62)	2.25	3.00	0.94(0.62)	2.02	2.75
Problems/Symptoms	1.33(0.66)	2.58	3.33	1.31(0.66)	2.53	3.33	1.00(0.60)	2.08	2.83	0.95(0.57)	1.92	3.50
Functioning	1.04(0.56)	1.92	2.67	1.04(0.58)	2.17	3.08	0.80(0.51)	1.84	2.33	0.70(0.50)	1.50	2.75
Risk	0.35(0.45)	1.18	3.00	0.30(0.47)	1.33	2.50	0.27(0.34)	1.00	1.67	0.15(0.27)	0.51	2.33
Non-risk items	1.17(0.57)	2.21	2.93	1.21(0.60)	2.26	2.89	0.90(0.49)	1.90	2.41	0.84(0.48)	1.68	2.71
All items	1.03(0.52)	2.00	2.88	1.05(0.55)	2.11	2.82	0.80(0.45)	1.71	2.47	0.72(0.43)	1.44	2.38

*SD* Standard deviation, *Max* Maximum score

In the pooled sample, all domains were negatively and significantly correlated with age, although with small to medium effect sizes. Coefficients ranged from −.14 for Risk to −.29 for Functioning. When looking only at the community subsample, age was significantly and negatively correlated with all domains except for Well-being (*ρ* = −.09). Correlations ranged from −.12 for Risk to −.14 for Functioning. In the student sample, correlations were statistically significant for all domains, except for Risk (*ρ* = −.08), and they ranged from −.12 for Problems/Symptoms to −.19 for Functioning.

Although these were not a priori planned analyses as requested by an anonymous reviewer we explored the differences of the scores with regard to the level of education (12 or less years of education vs. more than 12 years of education) for the community sample. In total 81 participants reported 12 or less years of education, while 227 reported more than 12 years of education. No significant differences in scores for the domain scores (95%CI for the mean difference of Well-being [−.07, .23], Problems [−.13, .16], Functioning [−.01, .27], and Risk [−.11, .03]), non-risk (−.06, .18) or total scores (−.05, .16) were identified between both groups.

### Correlations between domain scores

As expected, all domain scores were significantly associated with each other and with the total scores. The intercorrelations were strong between all domains except for Risk, which displayed low associations with all other scores (Table 6).

**Table 6:** Correlations between domains and with total scores for the pooled sample

Domains	Well-being	Problems	Functioning	Risk	Non-risk items
Problems	.71				
Functioning	.73	.69			
Risk	.41	.54	.48		
Non-risk items	.85	.92	.90	.54	
All items	.84	.92	.90	.60	1.00

## Discussion

This is the first study to explore the psychometric properties of the Spanish translation of the CORE-OM in Latin America. The results showed acceptable to good psychometric properties thus supporting the use of the Spanish version of the CORE-OM in the Ecuadorean population.

The acceptability of the measure (91.9% returned completed data) was good and comparable to that of the original UK version (91.0%), although somewhat lower than that reported in Spain (95.6%). The most omitted item (1.4%) in the present study, item 3 (“I have felt I have someone to turn to for support when needed”), was also the most omitted item in the Spanish population (0.7%).

The internal consistency for the all the items was excellent (*α* = .93) and comparable to both the UK version (*α* = .94) and that of the sample from Spain (*α* = .94). Non-risk items also had excellent internal consistency (*α* = .92). Regarding domain scores, internal consistency ranged from acceptable to good, with the exception of the domain with fewest items, Well-being (*α* = .69), for which it was borderline. Of note, the internal consistency for Well-being improved when considering only students (*α* = .74). This finding may reflect university students’ familiarity with the construct of well-being, as universities tend to promote and place an emphasis on student well-being and the relative newness of the well-being and psychological health generally in Ecuador, and particularly among less wealthy, less highly educated subpopulations. To the best of our knowledge, the CORE-OM well-being score alone has never been used as the only measure from the CORE-OM in any study, and any study aiming to separate the concept of well-being from other aspects of mental health and distress/dysfunction would be unwise to use only a four item scale. As noted above, the CORE-OM authors recommend only using individual domain scores as possible guides clinically where a client had problems mainly in one domain.

The results also showed support for test-retest stability again consistent with psychometric data reported in the UK and Spain [5, 19]. Lower stability in the Risk domain may relate to the volatile nature of the construct, as also suggested by Trujillo et al. [19]. Our data showed significant mean drop (decrease of psychological distress) from T1 to T2, but no significant change from the latter to T3. This pattern may be the often found effect of repeated test administration, as described by Durham et al. [31]. Given the general finding and these specific results, changes with only two administrations to non-clinical samples should be interpreted with caution.

There is evidence of good convergent validity for overall scores with both measures. The Risk domain presents the lowest convergent validity, congruent with all other studies of the CORE-OM and with the design expectation that the measure would not have a clean population factor structure. The OQ-45.2 contains “critical items” which focus on substance abuse in addition to self-harm, while the CORE-OM does not evaluate substance abuse. The SOS-10 does not include explicit risk items.

This study found a gender effect in the Well-being and Risk domains. Females showed lower levels of well-being, this effect size was small and consistent with results from the UK [5] and Spain [19]. In the Risk domain, males had higher scores than females, consistent with the UK results. Analyses of gender differences within subsamples revealed lower levels of Well-being for females in the student sample, and higher levels of Risk for males in the community sample. Although further research into these differences is needed, it seems possible that they will prove replicable and reflective of sociocultural issues, underscoring the need for locally pertinent referential data and for demographic variables, particularly gender, to be considered when interpreting scores.

Total and domain scores all exhibited a significant negative correlation with age, suggesting that psychological distress may diminish with age, though changes over time within individuals need not match cohort effects in cross-sectional data such as these. The cohort finding is consistent with prior research that indicates well-being is positively related with age in some countries [32].

Strong correlations between domains were expected because they all measure psychological distress. However, as noted [19], the Risk items, unlike the other 28 items, were designed act as flags for problematic behavior rather than for contribution to a general scale. This, and their lower variance than most other items, may contribute to the relative distinctness of Risk in terms of inter-domain correlations.

Strengths of the current study compared to the study from Spain [19] include a larger sample and participation of students from other majors in addition to psychology. The measurement of test-retest reliability through three time points in this study, as opposed to the more usual two, provides a broader assessment of temporal stability and mean shift. Lastly, the study followed on from a comprehensive qualitative and quantitative process to verify that the language of the translation conducted in Spain is understandable in an Ecuadorean context.

The nature of the present study was exploratory since no psychometric exploration of any psychotherapy change measure has been conducted in Ecuador at all. Limitations include the use of a convenience sample and resource limits meant the primary student participants were almost all contacted in Quito. Despite the snowballing outward, the overall sample frame was biased toward a higher than national average level of education was (more than 12 years) and had a relative lack of participants from rural areas. The absence of any population registry for the country made probability sampling impossible. However, when comparing the participants with more than 12 years of education, and those with 12 or less years of education, no significant differences were found in the scores. This would suggest that years of education is not associated with response to CORE-OM, though clearly more studies, and accumulation of a larger sample of persons with less than 12 years of education is needed to gain more precision for this finding. We have reported the 95th percentile for each score and each subsample to provide some guidance for interpretation of scores in non-clinical data but it is important that these statistics, particularly the 95th centiles, should be used with great caution pending collection of other and larger samples from Ecuador. As essentially no clinical services in Ecuador make routine use of outcome measures a clinical sample is accumulating only very slowly and not yet sufficiently large for analysis, hence cut-off sores could not be established.

## Conclusions

Despite the inevitable limitations, we believe the findings support the use of the CORE-OM as a valid and reliable measure for a non-clinical Ecuadorean population. Further studies with clinical samples are clearly necessary to provide cut-off scores and formal justification of use in a clinical context. However, it should be noted that to date no psychometric explorations of translations, or the original English, have shown marked psychometric differences between non-clinical and clinical populations other than, of course, the desired clinical/non-clinical differences on mean item, domain and total scores.

The fact that the measure can be used without a license fee, and the paucity of other free therapy change measures with local psychometric explorations, suggest that the CORE-OM is well suited for use in Latin America. Replication and extensions of this study, both in Ecuador and other countries, are needed and data collection is currently underway in collaboration with a number of other Latin American countries including Colombia, Peru, Chile and Uruguay, and with the Brazilian Portuguese translation, in Brazil. Researchers and clinicians from these and other Latin American countries are strongly encouraged to join this effort by contacting the first author.

## Data Availability

The datasets used and/or analyzed during the current study are available from the corresponding author on agreement about protection of confidentiality.

## Abbreviations

CORE-OM: Clinical Outcomes in Routine Evaluation-Outcome Measure
CST: CORE System Trust
UK: United Kingdom
OQ.45.2: Outcome Questionnaire 45.2
SOS-10: Schwartz Outcome Scale-10
CI: Confidence interval
SD: Standard Deviation
d1: Cohen’s d1 (mean change divided by the standard deviation)
dz.: Cohen’s dz (mean change divided by the standard deviation of the change values)
T1: Time 1
T2: Time 2
T3: Time 3
g: Hedges’ g
